# Audio-motor but not visuo-motor temporal recalibration speeds up sensory processing

**DOI:** 10.1371/journal.pone.0189242

**Published:** 2017-12-07

**Authors:** Yoshimori Sugano, Mirjam Keetels, Jean Vroomen

**Affiliations:** 1 Department of Industrial Management, Kyushu Sangyo University, Fukuoka, Japan; 2 Department of Cognitive Neuropsychology, Tilburg University, Tilburg, the Netherlands; Université catholique de Louvain, BELGIUM

## Abstract

Perception of synchrony between one's own action (a finger tap) and the sensory feedback thereof (a visual flash or an auditory pip) can be recalibrated after exposure to an artificially inserted delay between them (temporal recalibration effect: TRE). TRE might be mediated by a compensatory shift of motor timing (when did I tap?) and/or the sensory timing of the feedback (when did I hear/see the feedback?). To examine this, we asked participants to voluntarily tap their index finger at a constant pace while receiving visual or auditory feedback (a flash or pip) that was either synced or somewhat delayed relative to the tap. Following this exposure phase, they then performed a simple reaction time (RT) task to measure the sensory timing of the exposure stimulus, and a sensorimotor synchronization (SMS) task (tapping in synchrony with a flash or pip as pacing stimulus) to measure the point of subjective synchrony between the tap and pacing stimulus. The results showed that after exposure to delayed auditory feedback, participants tapped earlier (~21.5 ms) relative to auditory pacing stimuli (= temporal recalibration) and reacted faster (~5.6 ms) to auditory stimuli. For visual exposure and test stimuli, there were no such compensatory effects. These results indicate that adjustments of audio-motor synchrony can to some extent be explained by a change in the speed of auditory sensory processing. We discuss this in terms of an attentional modulation of sensory processing.

## Introduction

Precise and flexible control of action is of crucial importance for human behavior in everyday life. Especially timing is critical in actions like catching a ball, dancing, driving in traffic, or playing a musical instrument. Smooth action requires a proper order of movements with correct timings that can be learned with practice. However, even after these skills have been learned, they should be modifiable in order to adapt to rapid changes in environmental conditions [1, 2, 3] as well as to gradual change like growth in body size [4] that give rise to changes in neural transmission time. From this point of view, it can be argued that sensorimotor learning is a continuous recalibration process of when and how motor commands should be issued [1, 5, 6].

In order to study these adaptive changes in timing, one can introduce an artificial delay between an action and the sensory consequence (the feedback) of that action [7]. A study by Stetson et al. [8] demonstrated that perception of synchrony between a voluntary action and the feedback thereof is shifted after exposure to delayed sensory feedback. This shift is usually referred to as sensorimotor temporal recalibration effect (TRE) because the shift is, presumably, induced to reduce the timing error between events that normally co-occur. It has been shown that the sensorimotor TRE occurs for one's own delayed vocalization [9, 10], or viewing one's own delayed hand [11]. Furthermore, it has also been reported that it occurs if a visual stimulus precedes rather than follows a voluntary action [12, 13]. Thus, sensorimotor TRE is ubiquitous and robust phenomenon (for review, [2, 14]).

**Fig 1:**
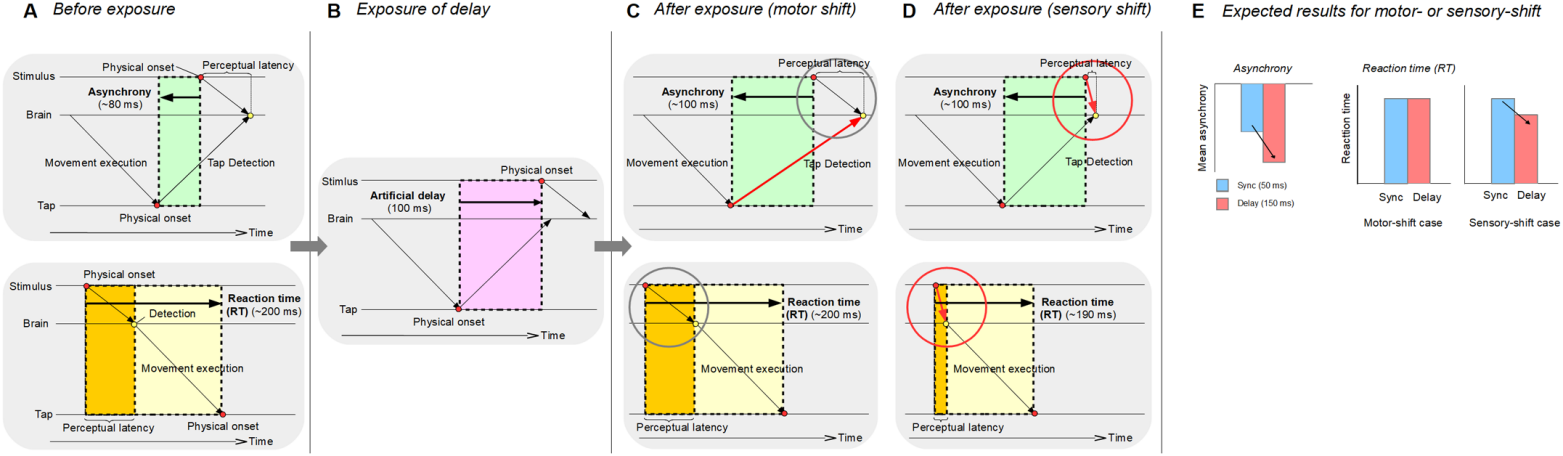
Predictions about changes in mean asynchrony and reaction time (RT). The model is adapted from Aschersleben and Prinz [21] with some modification. Each horizontal line represents a timeline for sensory timing, brain timing, and motor timing. Thin lines with an arrow that connects the three timelines represents a processing course between them. Distances among three timelines reflect processing latency. **(A)** Mean asynchrony (upper panel) and RT (lower panel) before exposure. **(B)** Exposure to delayed feedback during voluntary tapping. **(C, D)** Mean asynchrony and RT after exposure to delayed feedback: The perceived asynchrony might be reduced by a shift in the motor component **(C)** or the sensory component **(D)**. **(E)** Predictions about mean asynchrony and mean RT after exposure to synchronous or delayed feedback. Values in the figure are examples of real values to illustrate the direction of a shift.

Theoretically, sensorimotor TRE might be obtained via at least two different, though not mutually exclusive mechanisms: a change in motor timing ("when did I touch the surface?" or "when did I move my finger?"), or a change in the perceptual latency of the sensory feedback signal ("when did I hear the sound or see the flash"). Fig 1 illustrates how these shifts in perceived timing might induce a change in the perception of synchrony after exposure to delayed feedback. The mechanism underlying sensorimotor TRE is not clear. Previous researches suggest that sensorimotor TRE also occurs across sensory modalities (i.e., amodal) and might be controlled by rather central (cognitive) processes [15, 16, 17, 18, 19]. However, Sugano et al. [20] have shown that the magnitude of sensorimotor TRE is greater for audio-motor than visuo-motor pairings. And they also have shown that visuo-motor TRE transfers to audio-motor domain, but not vice versa. These results suggest that modality-specific mechanisms might be involved in the sensorimotor TRE.

TRE has also been demonstrated between pure sensory modalities like sound, vision and touch. These sensory adaptations to asynchronies have been found with artificial stimuli like flashes and beeps [22, 23, 24, 25, 26], and more natural stimuli like audio-visual speech [27, 28, 29]. Like sensorimotor TRE, both single amodal mechanism [30] and modality-specific mechanisms [31] are advocated so far.

Support for the existence of modality-specific mechanism comes from event-related potential (ERP) studies which have shown that the amplitude of early components in the ERP are modulated after exposure to visuo-motor delay [32] or audio-motor delay [33]. These results suggest that a modality-specific sensory shift might play a role during sensorimotor TRE. Furthermore, for audio-visual TRE, there is also evidence that perceptual latency of visual and/or auditory stimuli might change after exposure of audio-visual temporal asynchrony [31, 34, 35]. For example, after exposure to audiovisual asynchronies, Harrar and Harris [31] found a change in visual RT whereas Navarra et al. [34] reported a change in auditory RT. Di Luca et al. [35] reconciled this discrepancy by showing that visual RT changed if the audio-visual stimuli were presented from the same spatial position, whereas the auditory RT changed if the stimuli were not co-located. Note though that others reported that adaptation to audio-visual asynchrony does not depend on the spatial discrepancy of the audio-visual exposure stimuli [36], or on whether sounds were heard via speakers or headphones [22].

To date, there is no research that directly addresses whether perceptual latency changes following *sensorimotor* TRE. This was the motivation of the present research. In previous studies, it has been reported that tapping in synchrony with an external pacing signal (sensorimotor synchronization: SMS) provides a viable measure of sensorimotor TRE [17, 20, 37]. The common finding is that exposure to a fixed delay between a voluntary action (a finger tap) and an external sensory feedback signal (a flash or a tone) increases the natural anticipation tendency in a subsequent SMS task. Observers exposed to a delayed rather than synced feedback thus tap *earlier*, presumably to compensate the lingering effect of adaptation to the delay. Fig 1 shows a schematic illustration of how the timing of taps in the SMS task is modulated after delayed feedback. The model assumes that the movement execution time is not changed, as in principle it cannot compensate the delay. That is, even if a participant delays his/her movement execution to catch up the delayed feedback, it only results in delaying the feedback further. Rather, it is more likely that latency for tap detection or perceptual latency for stimulus is changed as it is effective for compensation (Fig 1C and 1D). Evidence from ERP studies support this assumption, because early modulations of visual and auditory evoked potential after exposure to delayed feedback have been demonstrated [32, 33].

The mean asynchrony is thought to correspond with the point of subjective simultaneity (PSS) between a tap and the pacing stimulus [20, 21, 38, 39, 40] (but see [41] for a different perspective of mean asynchrony. see [42] for review). However, with this task, one cannot tease apart a shift in the motor component (Fig 1C, upper) from a shift in the sensory component of the pacing stimulus (Fig 1D, upper). Therefore, we introduced a simple RT task along with the SMS task to separate the motor component from the sensory component. The rationale for using a simple RT task is as follows. Generally, a change in RT is thought to reflect a change in perceptual latency, but not a change in movement execution time [43, 44, 45, 46, 47, 48]. Specifically, as we assume that the movement execution time is not affected by the feedback delay, any change in RT should reflect a change in perceptual latency (Fig 1C and 1D). Moreover, earlier studies have shown that RT can be used to measure a change in perceptual latency after cross-sensory temporal recalibration [31, 34, 35]. It has also been suggested that the RT task and the temporal order judgement task are based on the same internal mechanism [49], thus giving further rationale of the use of RTs to measure a change in perceptual latency. We used the term "perceptual" and "sensory" as interchangeable here, because a simple RT task contains only two processes: the stimulus detection and the response execution (e.g., [50]). The stimulus detection is a low-level process that can be regarded as a sensory process.

It is of note that different interpretations of the mechanism underlying the asynchrony exist. Wohlschläger and Koch [41] suggests that the asynchrony is a result of a perceptual underestimation of the empty inter-onset intervals (IOIs). Also, others have suggested that periodic finger tapping is driven by an oscillatory motor activity that is coupled to a sensory driving oscillator (pacing stimuli) (e.g., [51]), in which the asynchrony can be a consequence of a phase lag caused by a detuning of coupled oscillators [52] (for review [42]). If it is the case that an exposure to delayed feedback further induces the underestimation of IOIs and/or further detunes the oscillatory motor activity from the sensory driving oscillator, it may give rise to a larger asynchrony than exposure to synchronous feedback. In fact, a slowing-down of the tapping tempo after exposure to delayed feedback is often observed in self-paced tapping (e.g., [53, 54]). As an aftereffect, it may lead to an acceleration of the tapping tempo during a subsequent SMS task and may give rise to an exaggeration of the asynchrony. Therefore, if one uses an SMS task to measure perception of synchrony, one should check if the inter-tap interval is not different across conditions during the SMS task.

One might suspect that the use of two different tasks to measure the same timing property, i.e., perceptual latency, is problematic because the SMS task relies on anticipation while the RT task relies on reaction. However, the SMS task contains a feedback-based error correction process of the asynchrony between tap and pacing stimulus (e.g., [42]), and so it involves a reaction to the current pacing stimulus. In the same vein, an RT task involves anticipation of the upcoming stimulus as is well-known from the foreperiod effect (e.g., [55]). Thus, in essence it appears that both tasks involve anticipation and reaction of upcoming stimuli.

One might also argue that the perceptual latency measured via an SMS task is beat-based, whereas latency measured via an RT task is non-beat-based. In fact, it has been shown that beat-based timing and non-beat-based timing involve different neural circuits (e.g. [56, 57, 58]). At present, we cannot say for certain if both tasks truly reflect the same timing property. However, we are optimistic regarding this for the following reasons. Firstly, the neural circuit for beat-based and the non-beat-based timing mechanism work in parallel via neural interconnections of the thalamus, pre-SMA/SMA, and the cerebral cortex [59]. Secondly, electrophysiological evidence has demonstrated that a visuo-motor TRE is accompanied by a change in early components of visual evoked potentials [32], and an early modulation of auditory evoked potentials accompanies a speed-up of auditory RT [60]. These findings might reinforce the validity of our methodology.

We had two motives in the present study. One was to test whether sensorimotor TRE contains a sensory component, and the second one was to examine whether this was different for the auditory than the visual modality. Regarding the first motive, we expected the mean asynchrony during the SMS task to increase (i.e., greater anticipation error) after exposure to delayed feedback. The extent to which this shift can be attributed to a shortening of the sensory component should be observable in the RT task. Here, we thus expected to observe faster RTs after delayed feedback that would diminish the artificial delay. Fig 1 shows a schematic illustration of how RTs might be modulated. If only the motor component shifts, then RTs do not change (Fig 1C, lower). However, if there is a shift in the sensory component (e.g., lowering the detection threshold), then RTs should become faster (Fig 1D, lower).

Regarding our second motive, consistent with our previous research [20, 37], we expected that the TRE would be greater following delayed auditory feedback than delayed visual feedback because sounds are also usually more potent to induce sensorimotor TRE. In general, audition is more precise than vision in temporal processing [3, 61, 62, 63, 64, 65]. Moreover, a stronger sensorimotor coupling has been suggested in audition than in vision [42, 66, 67]. This may lead to finer adjustment of sensory and/or motor timing in front of the delay than in vision, as the delay might be attributed to the timing error of the audio-motor system itself that should be adjusted [37].

## Method

### Participants

Seventeen students from Kyushu Sangyo University and one of the authors (Y.S.) participated in the experiment from November 2015 to January 2016 (one female, mean age 22.9 years ranged from 18 to 43 years, all were right-handed). All participants had normal hearing and normal or corrected-to-normal vision. Written informed consent was obtained from each participant. The experiment was approved by the Local Ethics Committee of Kyushu Sangyo University, and followed the declaration of Helsinki.

### Stimuli and apparatus

Participants sat at a desk in a dimly lit booth looking at a 17-inch CRT monitor running with 100-Hz refresh-rate at approximately 60 cm viewing distance. The visual stimulus was a 1 cm white square (30 ms duration, 9 cd/m^2^) with a black background (0 cd/m^2^) on the CRT monitor. The auditory stimulus was a 2000 Hz pure tone pip (30 ms duration with 2 ms rise/fall slope) presented via headphones (Sony MDR-CD900ST) at 79 dB(A). A 1-cm red square (30 ms duration, 3 cd/m^2^) and a 2250-Hz pure tone pip (30 ms duration with 2 ms rise/fall slope at 80 dB(A)) were used for catch trials (see Design and procedure). White noise was continuously presented via headphones at 60 dB(A) to mask the faint sound of mouse-presses. A special gaming mouse (Logitech G300) was used to obtain high temporal resolution (2 ms polling interval). Stimulus presentation and response detection were controlled by E-prime software running on a general PC/AT personal computer (Dell Precision T3400). The timing of stimulus presentation and response detection was verified by a multiple-trace oscilloscope.

### Design and procedure

Two within-subject factors were used: Exposure modality (visual vs. auditory) and exposure delay (50 ms vs. 150 ms). Exposure modality determined whether the feedback after a tap was auditory or visual, and exposure delay determined whether the sensory feedback was delayed (150 ms) or subjectively synchronous (50 ms). The trials for each of these 4 possible combinations were presented in blocks and these blocks were presented in counter-balanced order across participants.

**Fig 2:**
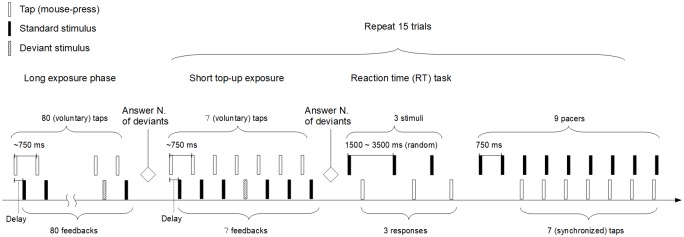
Experimental procedure for one block. Participants first experienced a long exposure phase (~1 min) in which they made 80 voluntary taps while receiving auditory or visual feedback (a pip or a flash) at delay of either 50 ms or 150 ms. Immediately after that, they received 15 test trials that each consisted of a short top-up exposure phase (7 voluntary taps with the same feedback as in the long exposure phase), a reaction time (RT) task in which they reacted as fast as possible to 3 pips or flashes, and a sensorimotor synchronization (SMS) task in which they tried to tap in synchrony with the last 7-out-of-9 pips or flashes. During the long exposure and the short top-up, participants counted the number of deviants (i.e., a red flash or a high-pitched tone) to ensure that they properly attend to the stimuli. At the end of the long exposure or the top-up, they reported the number of deviants.

Fig 2 shows a schematic illustration of the experimental procedure for a block of trials. Each block consisted of a long exposure phase (~1 min) followed by fifteen test trials that were each preceded by a short top-up exposure (~9 min in total). Each of the 4 possible blocks was presented once over 2 days. The exposure modality was the same during a day, and the exposure delay varied. There was a 5-min rest between blocks. The 2 days were separated by at least 24 hours (24 days maximum).

During the long exposure phase, participants made 80 voluntary mouse-presses without pacing stimuli with their index finger of the dominant hand, trying to maintain an inter-tap interval (ITI) of ~750 ms. Participants were instructed to maintain this tempo during the whole phase. Each mouse-press was followed by a sensory feedback (a flash on the CRT display or a tone via headphones, depending on the condition), delivered at either 50 ms (subjectively synchronous) or 150 ms (delayed). These values were chosen because they yielded consistent TRE in previous reports [17, 20, 37], and because the TRE of 150 ms is still in the range were subjects adapt to the delay (i.e., it becomes unnoticeable after a certain number of exposures). In order to ensure that participants pay attention to the modality in which feedback was delivered, they counted the number of deviants (i.e., a red flash or a high-pitched tone) during exposure. At the end of each exposure phase, they reported the number of deviants via a computer keyboard.

Following the long exposure phase, testing started. Each test trial consisted a short ‘top-up’ re-exposure phase (7 voluntary taps with the same feedback as in the long exposure phase), immediately followed by a simple RT task, and then followed by a SMS task. The order of the two tasks was chosen to minimize a possible carryover effect of the well-known slow-down in tapping tempo with delayed feedback (e.g., [53, 54]). During the RT task, participants were to press the mouse as quick as possible when they saw a flash or heard a click. Each stimulus was delivered three times at random ISIs between 1500 to 3500 ms. If the RT was shorter than 100 ms or longer than 700 ms, it was regarded as a mistake, and repeated to obtain at least one response.

Immediately after the RT task, participants then did the SMS task. During the SMS task, they tried to press a mouse in synchrony with an external pacing stimulus (the same flash or the tone as presented during the exposure phase). The pacing stimuli were delivered 9 times at a constant inter-stimulus interval (ISI) of 750 ms. Participants skipped the first two pacing signals to get into the rhythm, and then tried to sync their mouse-presses with the following 7 pacing stimuli. If an asynchrony (i.e., time difference between a pacing stimulus and a tap) was greater than +375 ms (the tap lagged the pacing stimulus by 375 ms) or smaller than -375 ms (the tap preceded the pacing stimulus by 375 ms), it was regarded as a mistake. If there were more than two mistakes in a trial, then the SMS task was repeated.

Participants had short practice session before the experiment to get accustomed to the experimental tasks. They did several test trials with feedback about their performance (i.e., tapping rate during the top-up exposure, mean asynchrony during the SMS task, and reaction time in the RT task), in which points were given depending on their performance. If they earned enough points, the practice session was finished. Then, they did extra three test-trials without performance feedback to be familiar with the main session. Testing lasted about 1.5 hour over two days.

## Results

### Mean RT and asynchrony

Trials from the practice session were excluded from further analysis. The asynchrony during the SMS task was defined as the difference in onset time between a tap and a pacing stimulus. A negative asynchrony means that the tap precedes the pacing stimulus (the well-known anticipation error, for review [42]). Missing responses and abnormal asynchronies (smaller than -300 ms or larger than +150 ms) were eliminated from analysis (0.7%). RT was measured from stimulus onset. Missing responses and abnormal RTs (shorter than 100 ms or longer than 450 ms) were also eliminated from analysis (3.0%). In addition to this screening, asynchronies and RTs that were outside 1.5 times the interquartile range below or above the appropriate quartile were also treated as outliers on a per-participant basis, and they were also eliminated from the analysis (2.4% for asynchrony and 5.8% for RT).

TAs were averaged over trials for each experimental condition and each participant. The RT distribution was positively skewed, and median instead of mean RT was calculated. Table 1 shows the group-averaged mean asynchrony and RT for each experimental condition. The individual data were entered into a repeated-measures ANOVA with exposure modality (MV vs. MA) and exposure delay (sync vs. delay) as within-subjects factors.

**Table 1:** Mean asynchrony and simple RT in milliseconds for each experimental condition.

Modality	Exposure delay	Mean asynchrony (ms)	Simple reaction time (ms)
Motor-auditory (MA)	Sync (50 ms)	-87.5 (9.8)	179.1 (3.4)
	Delay (150 ms)	-109.0 (9.8)	173.6 (3.7)
	Delay—Sync	-21.5 ***	-5.6 *
Motor-visual (MV)	Sync (50 ms)	-55.9 (9.6)	191.4 (3.7)
	Delay (150 ms)	-55.8 (8.0)	190.5 (4.3)
	Delay—Sync	0.1 n.s.	-0.9 n.s.
Mean-MA—Mean-MV		-42.4 ***	-14.6 ***

Note:

* p < 0.05,

*** p < 0.001.

SEMs are shown in parenthesis. Simple reaction times were tested on reciprocally transformed values.

As the distribution of median-RTs was significantly different from normal (Shapiro-Wilk normality test, W = 0.96, p < 0.05), a reciprocal transformation was applied. After this transformation, the distribution was not significantly different from normal (W = 0.98, p = 0.232). These transformed RTs were used in all statistical tests. Although it should be better to report back-transformed values of the mean of transformed RTs in Table 1 (see [68], p.338), we reported simple mean of RTs instead, because it is convenient. Also, the two values were highly correlated (r ~ = 1.0, p < 0.001) and thus provided the same conclusion.

The ANOVA on the asynchronies revealed significant effects of exposure modality, F(1, 17) = 26.0, p < 0.001, exposure delay, F(1, 17) = 11.9, p < 0.01, and an interaction of the two, F(1, 17) = 17.2, p < 0.001. The first one indicates that the mean asynchrony was more negative in MA (-98.3 ms) than MV (-55.9 ms), which is the usual pattern in this task (e.g., [17, 20, 37, 42]). In order to examine the interaction, the asynchronies were analyzed separately for each exposure modality and were entered into a repeated-measures ANOVA with exposure delay (sync vs. delay) as a within-subjects factor. These ANOVAs revealed that the effect of exposure delay was significant for the MA condition, F(1, 17) = 46.8, p < 0.001 (the mean asynchrony was more negative (~21.5 ms) after exposure to delayed auditory feedback than synced one: -109.0 ms vs. -87.5 ms), but not for the MV condition, F(1, 17) < 0.001, p = 0.989 (the mean asynchrony was not significantly different (~0.1 ms) after exposure to delayed visual feedback from synced one: -55.8 ms vs. -55.9 ms).

As we mentioned in the introduction, there is a possibility that the observed TRE may be exaggerated under the delayed feedback condition by an acceleration of tapping tempo during the SMS task. If this is indeed the case, the mean asynchrony would progressively increase with taps. However, we did not find such a tendency by running the following additional analysis. Repeated-measures ANOVAs per modality on the mean asynchronies with exposure delay and taps as within-subjects factors showed that exposure delay x tap interaction was not significant in both MV, F(6, 96) = 0.49, p = 0.814, and MA, F(6, 96) = 1.23, p = 0.296, meaning the size of the TRE did not change across taps. Thus, we believe that the exaggeration of TRE might be negligible, if any.

The ANOVA on the RTs revealed a significant effect of exposure modality, F(1, 17) = 18.0, p < 0.001, and it interacted with exposure delay, F(1, 17) = 4.8, p < 0.05. The mean RT was significantly shorter (~14.6 ms) for auditory (176.3 ms) than visual stimuli (191.0 ms), which is in line with earlier findings (e.g., [69, 70, 71]). In order to examine the interaction, RTs were separated by exposure modality, and entered into a repeated-measures ANOVA with exposure delay (sync vs. delay) as a within-subjects factor. These ANOVAs revealed that the effect of exposure delay on the RT was significant for the MA condition (the mean RT was ~5.6 ms faster after exposure to delayed than synced auditory feedback: -179.1 ms vs. 173.6 ms), F(1, 17) = 5.6, p < 0.05, but not for the MV condition (no significant difference (~0.9 ms) in the mean RT after delayed versus synced visual feedback: 191.4 ms vs. 190.5 ms), F(1, 17) = 0.7, p = 0.414.

To further analyze RTs, we fitted an ex-Gaussian distribution on the response time distribution [55, 72, 73, 74, 75, 76, 77]. The ex-Gaussian is made of a convolution of a Gaussian with an exponential function that is characterized by three parameters: mu, sigma, and tau, which are thought to reflect different perceptual and/or cognitive processes [55, 76, 77]. For each participant, we thus obtained estimates of mu, sigma, and tau. These estimates were then entered into repeated-measures ANOVAs per exposure modality with exposure delay (sync vs. delay) as a within-subjects factor. As the distribution of mu and tau were significantly different from normal (Shapiro-Wilk normality test; W = 0.96, p < 0.05; W = 0.92, p < 0.001; respectively), the mus were applied by a reciprocal transformation and the taus were applied by square-root transformation before ANOVA. The analyses showed a main effect of exposure-delay on the tau of MA-RT (14.7 ms for delayed feedback vs. 20.2 ms for synced feedback), F(1,17) = 14.0, p < 0.01.

**Fig 3:**
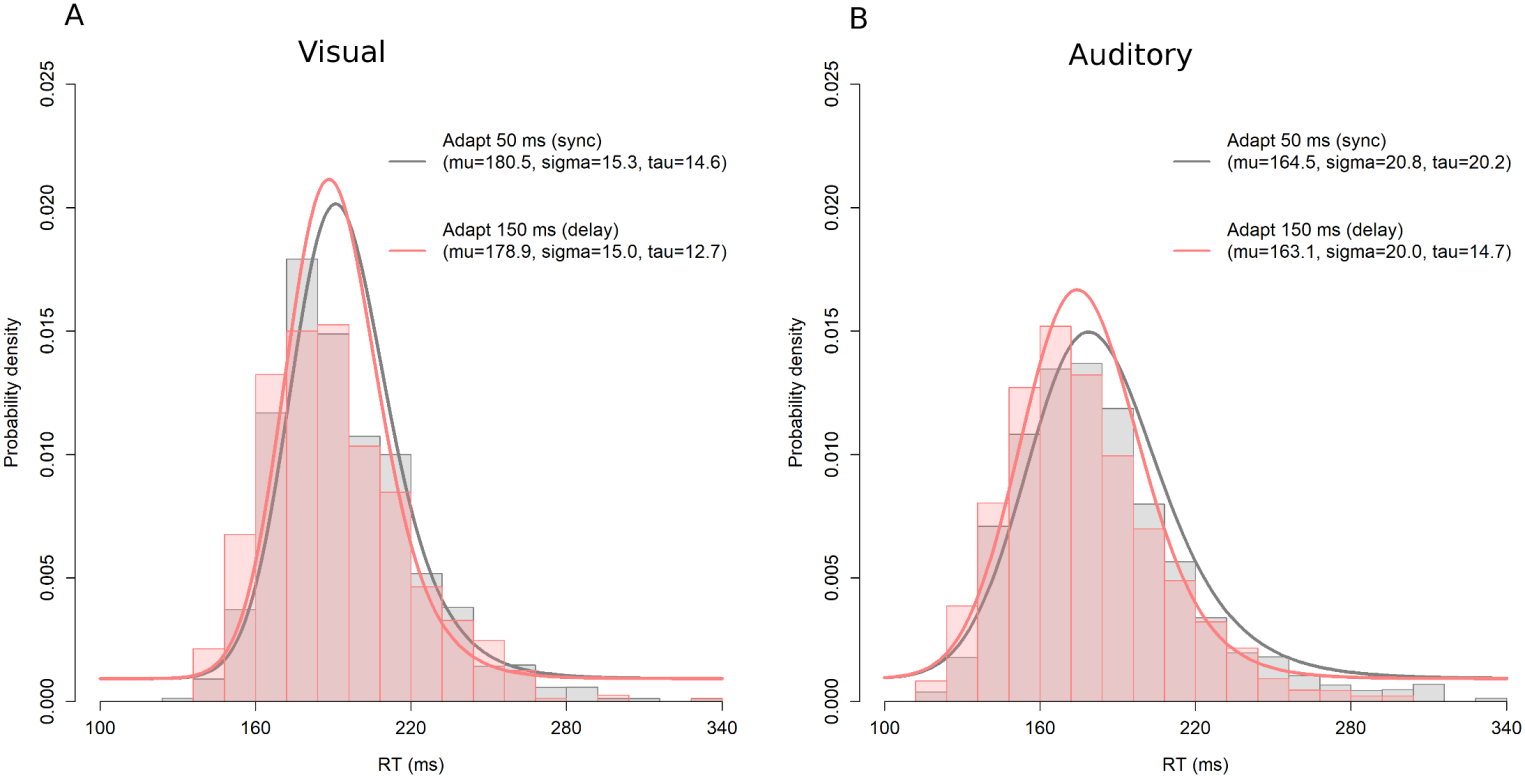
Group-averaged histograms and group-averaged ex-Gaussian functions that are fitted to reaction times for each participant and each experimental condition. **(A)** Visual RT, and **(B)** Auditory RT. Estimated parameters of a fitting curve are also shown.

Fig 3 shows the group-averaged histograms and the group-averaged ex-Gaussian functions to fit the reaction times for each participant and each condition. As is clearly visible, only for MA-RTs (Fig 3B) tau became smaller after exposure to delayed rather than synced feedback (14.7 vs. 20.2 ms, respectively), while mu (163.1 vs. 164.5 ms) and sigma (20.0 vs. 20.8 ms) were unchanged. As the tau parameter mainly affects the tail of the distribution, smaller tau means that slower RTs were sped-up. Exposure to delayed auditory feedback thus induced larger anticipation responses in tapping and faster RTs, whereas exposure to delayed visual feedback did not change these measures.

### Variability of RT and asynchrony

It has been suggested that the sensitivity to temporal asynchrony between different sensory modalities (i.e., sound and light) is lowered in the initial stages of the build-up of TRE [24, 78, 79]. The temporal window of crossmodal integration is thus first widened to accommodate a discrepancy of timing (see also [80]). To examine this, we analyzed the variability in SMS and RTs after exposure to delayed feedback. We assumed that tapping variability is inversely correlated with the sensitivity of timing (the less sensitive, the more variable tapping is) [42, 81, 82].

We calculated a mean within-trial standard deviation (WSD) of asynchrony as a measure for variability for each participant, which is defined as an averaged within-trial standard deviation of asynchrony over trials. In the following all statistical tests, they were given logarithmic transformation to let them normally distributed. For RTs, as there was only three RTs within one trial, we calculated a quartile deviation (QD) over all RTs. The group-averaged asynchrony-WSDs and RT-QDs are shown in Table 2.

**Table 2:** Mean within-trial standard deviation (WSD) of asynchrony and mean quartile deviation (QD) of simple reaction time (RT) in milliseconds for each experimental condition.

Modality	Exposure delay	Mean asynchrony (ms)	Simple reaction time (ms)
Motor-auditory (MA)	Sync (50 ms)	29.1 (1.6)	18.4 (1.2)
	Delay (150 ms)	30.7 (1.7)	16.9 (1.1)
	Delay—Sync	1.6 n.s.	-1.5 n.s.
Motor-visual (MV)	Sync (50 ms)	39.7 (3.2)	14.4 (1.1)
	Delay (150 ms)	36.8 (2.5)	13.4 (1.1)
	Delay—Sync	-3.0 n.s.	-1.0 n.s.
Mean-MA—Mean-MV		-8.4 ***	3.8 **

Note:

** p < 0.01,

*** p < 0.001.

SEMs are shown in parenthesis. QD of simple reaction times were calculated over all RTs for each participant. WSD of asynchrony was tested on logarithmically transformed values.

Mean asynchrony-WSDs and RT-QDs were entered into a repeated-measures ANOVA with exposure modality (MV vs. MA) and exposure delay (sync vs. delay) as within-subjects factors. The ANOVA on the asynchrony-WSDs revealed that the main effect of modality was significant, F(1, 17) = 38.3, p < 0.001, as well as the delay x modality interaction, F(1, 17) = 9.9, p < 0.01. The main effect of exposure modality indicated that asynchrony-WSD was significantly larger in MV (38.2 ms) than MA (29.9 ms). Subsequent repeated-measures ANOVAs per modality with exposure delay as within-subjects factor revealed that the effect of exposure delay was not significant in both MA, F(1, 17) = 2.4, p = 0.139, and MV, F(1, 17) = 2.2, p = 0.160. The ANOVA on the RT-QDs, on the other hand, revealed that the main effect of modality was significant, F(1, 17) = 9.0, p < 0.01, indicating that it was significantly larger in MA (17.7 ms) than MV (13.9 ms).

## Discussion

Our first motive was to examine whether adjustments in the perception of motor-sensory synchrony involve shifts in sensory timing. The TRE may contain both a shift of sensory timing and a shift of motor timing (Fig 1). However, to date, there is no research that directly addresses to what extent the sensory shift contributes to the TRE. We used a simple RT task to estimate the perceptual latency, and used a SMS task to estimate the size of TRE.

Our second motive was to examine if the TRE was different for auditory versus visual feedback. Temporal processing is usually more precise in audition than vision, and there might be stronger audio-motor than visuo-motor connections, and so we predicted a stronger MA-TRE than MV-TRE.

In order to examine this, participants tapped their index finger at a constant pace while receiving visual or auditory feedback (a flash or pip) that was either synced with the tap or somewhat delayed. Following an exposure phase, they then performed a simple RT task to measure the sensory timing of the exposure stimulus, and a SMS task to measure the point of subjective synchrony between finger taps and the pacing stimuli. The results showed that after exposure to delayed auditory feedback, participants reacted faster (~5.6 ms) to auditory stimuli and tapped earlier (~21.5 ms) relative to auditory pacing stimuli (= MA-TRE). For visual exposure and test stimuli, there was no difference after exposure to synced versus delayed feedback in RTs and tapping. Taken together, these results thus show, for the first time, that recalibration of audio-motor synchrony can partly be explained by a change in the speed of auditory sensory processing. This result fits previous reports on audio-visual temporal recalibration that also reported that exposure to audio-visual asynchrony is accompanied by shifts in auditory latency [34, 35].

Our results are also consistent with previous finding about auditory dominance over vision in sensorimotor TRE. In general, sounds appear to be more potent than visual stimuli to induce (or erase) temporal recalibration. For example, MV-TRE following exposure to delayed visual feedback is erased when it is mixed with synchronous auditory feedback [37]. Our findings can also explain our earlier finding that MV-TRE transfers to the MA domain, but that MA-TRE does not transfer to the MV domain. No transfer of the MA-TRE to MV domain would imply that the MA-TRE is mediated by a shift of sensory component (change in perceptual latency), whereas the MV-TRE might be mediated by a more amodal mechanism or a shift of movement-related timing [20].

### Speed-up of auditory RT

An important question is how a speeding-up of perceptual latency can accompany sensorimotor TRE. For *cross-sensory* TRE, several mechanisms have been proposed like: (1) adjustment of a criterion for synchrony, (2) adjustment of a sensory threshold, (3) a widening of the temporal window for cross-sensory integration [2, 14], and (4) adaptation of neuronal population to specific temporal intervals [83, 84]. In principle, all these models can be applied to *sensorimotor* TRE as well, but among these models, only the threshold adjustment model makes clear predictions about a change in perceptual latency (see also [35]). If so, then how can a sensory threshold be adjusted? In the next sections, we discuss several possible mechanisms by which a sensory threshold can be adjusted.

One potential mechanism to cause an adjustment of sensory threshold could be an attentional shift in time [85, 86, 87, 88]. If a participant notices a delay between an action and a sensory feedback thereof, attention may be reallocated to the delayed feedback as it indicates that something unnatural occurs. Indeed, it has been shown that if a sound is presented at irregular timings within a regular sound sequence, it automatically captures one's attention [89]. Moreover, there is a robust evidence showing that our sensorimotor system can detect subliminal (i.e., cannot be noticed) timing perturbation of regular sequence of sound during SMS [90]. In addition, attention to a sensory stimulus can enhance the processing time of that stimulus (the law of prior entry [91, 92]; see [2] for review). Both behavioural (e.g., temporal order judgement (TOJ) task) and electrophysiological studies (e.g., event-related potential response: ERP) have shown that an attended sensory stimulus is indeed processed faster [92, 93, 94, 95], and delayed sensory feedback might thus capture attention, thereby speeding-up the sensory processing of that stimulus [35]. This hypothesis also fits with electrophysiological studies that have shown that delayed auditory feedback after one's own action elicits enhanced neural responses (P2 and N300), that is correlated with one's awareness of that delay [96]. These authors have suggested that the enhanced P2 is related to attention to the delayed auditory stimuli.

The analysis by ex-Gaussian fitting to RT distribution gives additional support of the attentional shift explanation. The tau of MA-RT was significantly smaller for delayed feedback (14.7 ms) than for synced feedback (20.2 ms). As the tau parameter is thought to reflect more central, attention demanding (analytic) processes [77, 97, 98] (but see [76]), this also favors the attentional shift explanations. Harrar et al. [77] reported a similar result in which both tau and sigma of unisensory RT distribution were changed after exposure to audio-visual asynchrony.

A weak point of this attentional explanation is that if attentional resources were allocated more to delayed than synced feedback, delayed feedback would also have a detrimental effect on the movement control, because attention for temporal features are thought to be shared with the execution of a movement [99]. This might result in more variable (i.e., less controlled) tapping in the delayed feedback condition than in the synced condition ([80] for similar discussion). Earlier study about sensorimotor TRE indeed showed this pattern [37], but not in the current study. In order to examine this further, future studies might instruct participants to attend to their own movement (i.e., tap) or to the feedback (i.e., flash or tone) and then measure whether a speed-up of RT is obtained irrespective of attentional direction.

It is of interest to note that shifts in perceived timing have also been reported in the literature on *intentional binding* [80]. Intentional binding refers to the phenomenon that the timing of a voluntary action and the sensory feedback thereof attract each other in time. In the study of Haggard [80], participants were asked to press a key at a time of their own choice, which triggered a beep after 250 ms. Participants were asked to judge the perceived time of their keypress or of the beep relative to a rotating clock. Result showed that the perceived time of the action shifted 15 ms (6% shift) later in time (towards the beep), and the perceived time of the beep shifted 46 ms (18.4% shift) earlier in time (towards the action). Although the relationship between intentional binding and sensorimotor TRE still remains elusive [12], a lot of similarities have been reported so far. For example, both decrease if the feedback comes several hundreds of milliseconds after the action (e.g., ~400 ms [8, 15, 80], but see [100, 101] who reported that intentional binding still occurs as long as 4 sec). Both are tightly connected to perception of causality between action and feedback [13, 102] and to a sense of agency [13, 103]. Both occur across various sensory modalities [15, 16, 104]. Both occur even if the sense of body ownership is weak [11, 105, 106]. Finally, both occur even if the feedback signal comes *before* the action [12, 107] (but see [13] who have shown that if TRE was measured by interval estimation, it was limited to the movement-lead side). Considering these similarities, intentional binding and TRE might reflect the same perceptual and/or cognitive process: *temporal grouping*. Intentional binding reflects the immediate perceptual bias in time, and sensorimotor TRE is the aftereffect of it [8, 108]. However, some insisted the opposite relationship: the sensorimotor TRE is underlying mechanism that *causes* intentional binding [109]. More research is needed to elucidate the relationship between intentional binding and sensorimotor TRE in future.

### Auditory dominance in sensorimotor TRE

Why is it that auditory, but not visual delayed feedback induced TRE and sped-up RTs? (see also [15, 20, 37, 110]). One factor might be that auditory stimuli have more power to capture one's attention than visual stimuli [111, 112, 113, 114, 115], and possibly attention is also of importance in TRE [116, 117, 118]. For example, Heron et al. [116] argued that attention to the feedback delay enhances audio-visual TRE. Ikumi and Soto-Faraco [117] has shown that if participants were exposed to staggered audio-visual sequences, the direction of TRE depended on whether participants attended to AV grouping or VA grouping. Tsujita and Ichikawa [118] reported that awareness to a visual delay is even necessary for visuomotor TRE to occur.

Furthermore, temporal resolution is better for the auditory modality than the visual one [37, 61, 62, 63]. Such a superiority of temporal processing of audition over vision could lead to a lower delay-detection threshold in audition than vision [16, 119], resulting in auditory dominance over vision in sensorimotor TRE, which might imply that sensorimotor TRE can be affected by a top-down factor such as an attentional shift.

It might also be that there is a stronger coupling of perception and action in the MA domain than in the MV domain. It has been suggested that the auditory system is more strongly coupled to the motor system than the visual system [120, 121, 122]. Strong audio-motor coupling could then produce stronger "intentional binding" [80] for MA than MV. However, this was not the case in data reported by Engbert et al. [104], who found that the amount of compression between action and feedback by intentional binding was equivalent across audio-motor, visuo-motor and motor-tactile pairs. However, they reported a modality effect where the estimated interval was shorter for audio-motor than visuo-motor or motor-tactile interval. They interpreted this modality effect as "the interval prior to auditory stimulus is… retrospectively compressed in subjective time" (p.700). It might reflect a strong coupling between movement and auditory processing [42, 66], which is based on strong neural connectivity between motor cortex and auditory cortex [67].

Unlike earlier studies [20, 37, 40], we failed to obtain a reliable MV-TRE. One possibility for the absence of MV-TRE is that performing the MA condition erased the subsequent MV-TRE, even though they were separated by at least 24 hours. Indeed, Sugano et al. [37] have shown that preceding MA execution erased the subsequent MV-TRE if MA and MV were alternated within one experimental session. To check this possibility, we did an additional mixed-model ANOVA on MV-asynchrony with the order of modality (MA first vs. MV first) as a between-subjects factor and the exposure delay as a within-subjects factor. This ANOVA showed no significant effect of modality order, F(1, 16) = 1.9, p = 0.190, nor an interaction of two factors, F(1, 16) = 0.59, p = 0.455, indicating that the execution order of modality did not affect the size of MV-TRE (i.e., difference of MV-asynchrony between delay and sync). The absence of the MV-TRE thus cannot be explained by erasure via the preceding MA condition. A more likely possibility might be that the MV-TRE was so fragile that it did not survive the intervening RT task (which preceded the SMS task). This fits data from a pilot study of ours [123] in which we did obtain a significant MV-TRE (16.3% shift of mean asynchrony between sync and delay) under the procedure that the order of SMS and RT task was reversed (i.e., the SMS task preceded the RT task). In the pilot study, MA-TRE was also bigger (33.4% shift) than the present study, and the auditory RT was significantly shorter by 6.5 ms after exposure to motor-auditory delays.

### Cue reliability and predictability

The reliability of timing information in the motor, visual, and auditory domains should affect TRE [34, 35]. In general, the modality that conveys more reliable information dominates the other modality [124, 125, 126] (for review, [14, 127]). It is also well-known that auditory perception, in general, has a finer temporal resolution than visual perception [3, 61, 62, 63, 64, 65], and this was also found here in that the tapping variability in MA was smaller than in MV (~29.9 ms in MA versus ~38.2 ms in MV).

With this in mind, one might expect that visual timing is more malleable than auditory timing. However, the present results indicated that the shift in motor timing was largest (it was estimated by subtracting the shift of perceptual latency (5.6 ms shift in the MA) from the total size of TRE (e.g., 21.5 ms shift in the MA), which gave 15.9 ms motor-shift in the MA), followed by auditory timing (which is reflected by the RT shift, a 5.6 ms shift), whereas the shift of visual timing was smallest (0.9 ms shift) (see Table 1).

A key concept that might reconcile this discrepancy is the predictability of when a sensory signal is to occur. If the timing of a sensory signal is more predictable, the more adaptable it could be. Rohde et al. [40] found support for this explanation and argued that predictability is necessary for visuomotor TRE to occur. Moreover, Vercillo et al. [128] showed that inaccurate perception of timing prevents sensorimotor TRE to occur. Supporting evidence also comes from studies of the intentional binding. It is well known that predictability of sensory feedback enhances the intentional binding [80, 129]. Perhaps, whether or not events are controllable might be more important for sensory-motor TRE than the cue reliability, which might make predictability play a key role in sensory-motor TRE [40].

### Variability of asynchrony and RT

The non-significant difference in variability of asynchrony between sync and delay is in line with our earlier study [17, 20] (but see [37]). It supports the notion that the sensitivity to temporal asynchrony does not change after exposure to delayed sensory feedback. Lesser tapping variability in MA than MV was also compatible to earlier findings indicating that tapping is more stable for auditory pacing stimuli than visual pacing stimuli [20, 37, 121, 122, 130] (for review [42]). However, the greater variability in MA-RT than MV-RT is not in line with earlier researches, which showed that visual RT is more variable than auditory one [46, 131, 132]. One possible explanation might be that the perceived strength of auditory stimuli is weaker than that of visual stimuli in the present study (e.g., additional white noise during the experiment masks auditory stimuli), which causes more variable RT in MA than in MV [46].

## Conclusion

Our results demonstrate that delayed auditory feedback to finger taps not only induced a shift in the perception of audio-motor synchrony, but it also sped-up auditory latency that (partly) compensated the delay. Delayed visual feedback did not induce similar shifts in the perception of motor-visual synchrony or visual latency, presumably because timing information in the visual domain is less precise and salient. These results thus demonstrate the brain maintains unity between the senses partly by shifting them in time.

## Supporting information

S1 Dataset CSV files for asynchronies and RTs in the present study. Raw data, processed raw data in which outliers are eliminated, and mean (median) and WSD (QD) of asynchrony and RTs are included. See 'README.txt' for descriptions of each CSV file.
(ZIP)
